# Membrane Protein Amuc_1100 Derived from Akkermansia muciniphila Facilitates Lipolysis and Browning *via* Activating the AC3/PKA/HSL Pathway

**DOI:** 10.1128/spectrum.04323-22

**Published:** 2023-02-27

**Authors:** Xifen Zheng, Wenting Huang, Qianbei Li, Yun Chen, Linyan Wu, Yifan Dong, Xinyue Huang, Xiaojing He, Zihao Ou, Yongzheng Peng

**Affiliations:** a Department of Laboratory Medicine, Zhujiang Hospital, Southern Medical University, Guangzhou, China; b Department of Transfusion Medicine, Zhujiang Hospital, Southern Medical University, Guangzhou, China; c Department of Laboratory Medicine, Nanfang Hospital, Southern Medical University, Guangzhou, China; d Department of Gynaecology and Obstetrics, Nanfang Hospital, Southern Medical University, Guangzhou, China; Shangdong First Medical University

**Keywords:** Amuc_1100, lipolysis, browning, AC3/PKA/HSL pathway, preadipocytes

## Abstract

Obesity, defined as a disorder of lipid metabolism caused by white fat accumulation, is closely related to the gut microbiota. Akkermansia muciniphila (Akk), one of the most common gut commensals, can reduce fat storage and promote the browning of white adipocytes, alleviating disorders of lipid metabolism. However, which components of Akk produce the effect remain unclear, limiting the application of Akk in the treatment of obesity. Here, we found that the membrane protein Amuc_1100 of Akk decreased formation of lipid droplets and fat accumulation during the differentiation process and stimulated browning *in vivo* and *in vitro*. Transcriptomics revealed that Amuc_1100 accelerated lipolysis through upregulation of the AC3/PKA/HSL pathway in 3T3-L1 preadipocytes. Quantitative PCR (qPCR) and Western blotting showed that Amuc_1100 intervention promotes steatolysis and browning of preadipocytes by increasing lipolysis-related genes (*AC3*/*PKA*/*HSL*) and brown adipocyte marker genes (*PPARγ*, *UCP1*, and *PGC1α*) at both the mRNA and protein levels. These findings introduce new insight into the effects of beneficial bacteria and provide new avenues for the treatment of obesity.

**IMPORTANCE** An important intestinal bacterial strain Akkermansia muciniphila contributes to improving carbohydrate and lipid metabolism, thus alleviating obesity symptoms. Here, we find that the Akk membrane protein Amuc_1100 regulates lipid metabolism in 3T3-L1 preadipocytes. Amuc_1100 inhibits lipid adipogenesis and accumulation during the differentiation process of preadipocytes, upregulates the browning-related genes of preadipocytes, and promotes thermogenesis through activation of uncoupling protein-1 (*UCP-1*), including Acox1 involved in lipid oxidation. Amuc_1100 accelerates lipolysis *via* the AC3/PKA/HSL pathway, phosphorylating HSL at Ser 660. The experiments illustrated here identify the specific molecules and functional mechanisms of Akk. Therapeutic approaches with Amuc_1100 derived from Akk may help alleviate obesity and metabolic disorders.

## INTRODUCTION

Globally, the prevalence of obesity and being overweight continues to rise, and more than 710 million people are considered obese (1, 2). Obesity causes a series of related diseases, such as hyperlipidemia, type 2 diabetes, cardiovascular disease, nonalcoholic fatty liver disease, and tumors (3–5). There are three types of adipocytes in the human body: white adipocytes, which can store energy as fat within a single large cellular lipid droplet; brown adipocytes, which are rich in mitochondria, express high levels of uncoupling protein-1 (*UCP-1*), and are characterized by the ability to burn energy during nonshivering thermogenesis, and beige or brite (brown in white) adipocytes, which are intermediates between white and brown adipocytes (6, 7). Obesity is characterized by abnormal accumulation of white adipocytes and the relative reduction of brown adipocytes in the body (8). White adipocytes have the potential to convert to brown-like adipocytes, which is called “browning,” a process that helps to consume excess energy and maintain energy balance *in vivo* (9, 10). Therefore, it is critical for obesity treatment to explore a safe and effective way of inducing the browning of white adipocytes.

Changes in the composition and function of gut microbiota affect energy metabolism, insulin sensitivity, thermogenesis in brown adipocytes, and the “browning” of white adipocytes (11–13). An important intestinal bacterial strain Akkermansia muciniphila (Akk) can relieve insulin resistance, induce partial white adipocyte browning, and increase energy expenditure, thus alleviating obesity symptoms (14–16). Akk holds significant promise for the treatment of obesity, but the use of live biotherapeutics was found to cause septicemia in compromised patients (17), detailing the possible risks of fecal microbiota transplantation (FMT) with live Akk in obesity treatment. Therefore, identifying the specific molecules and functional mechanisms of Akk are of great significance for ensuring the safe and effective clinical application of Akk.

Amuc_1100, one of the most abundant pili-like proteins expressed on the outer membrane of Akk, produces most of the beneficial effects of the bacteria (18). Previous studies have demonstrated that the effect of Akk on host lipid metabolism may be related to its outer membrane protein Amuc_1100 (19, 20). However, the regulation mechanism for Amuc_1100 has not yet been elucidated. In the current study, our experiments showed that Amuc_1100 inhibits adipogenesis and accumulation of lipids during the differentiation process *in vitro* and *in vivo*. Additionally, Amuc_1100 upregulates the browning-related genes (peroxisome proliferator-activated receptor γ [*PPAR-γ*], coactivator 1α [*PGC1α*], and *FGF21*) of 3T3-L1 preadipocytes and promotes the activity of *UCP-1* in mitochondria, including *Acox1*, which is involved in β-oxidation of fatty acids. Transcriptomics analysis identified that Amuc_1100 accelerates lipid decomposition through upregulation of the AC3/PKA/HSL pathway in 3T3-L1 preadipocytes. Western blotting indicated that Amuc_1100 increases the phosphorylation of hormone-sensitive lipase (*HSL*) at the Ser 660 site. In conclusion, Amuc_1100 effectively promotes preadipocyte browning and energy consumption *via* thermogenesis from lipolysis, providing a strong foundation for the development and clinical application of Akk-related treatment of obesity.

## RESULTS

### Amuc_1100 attenuates high-fat diet (HFD)-induced adiposity *in vivo*.

**FIG 1:**
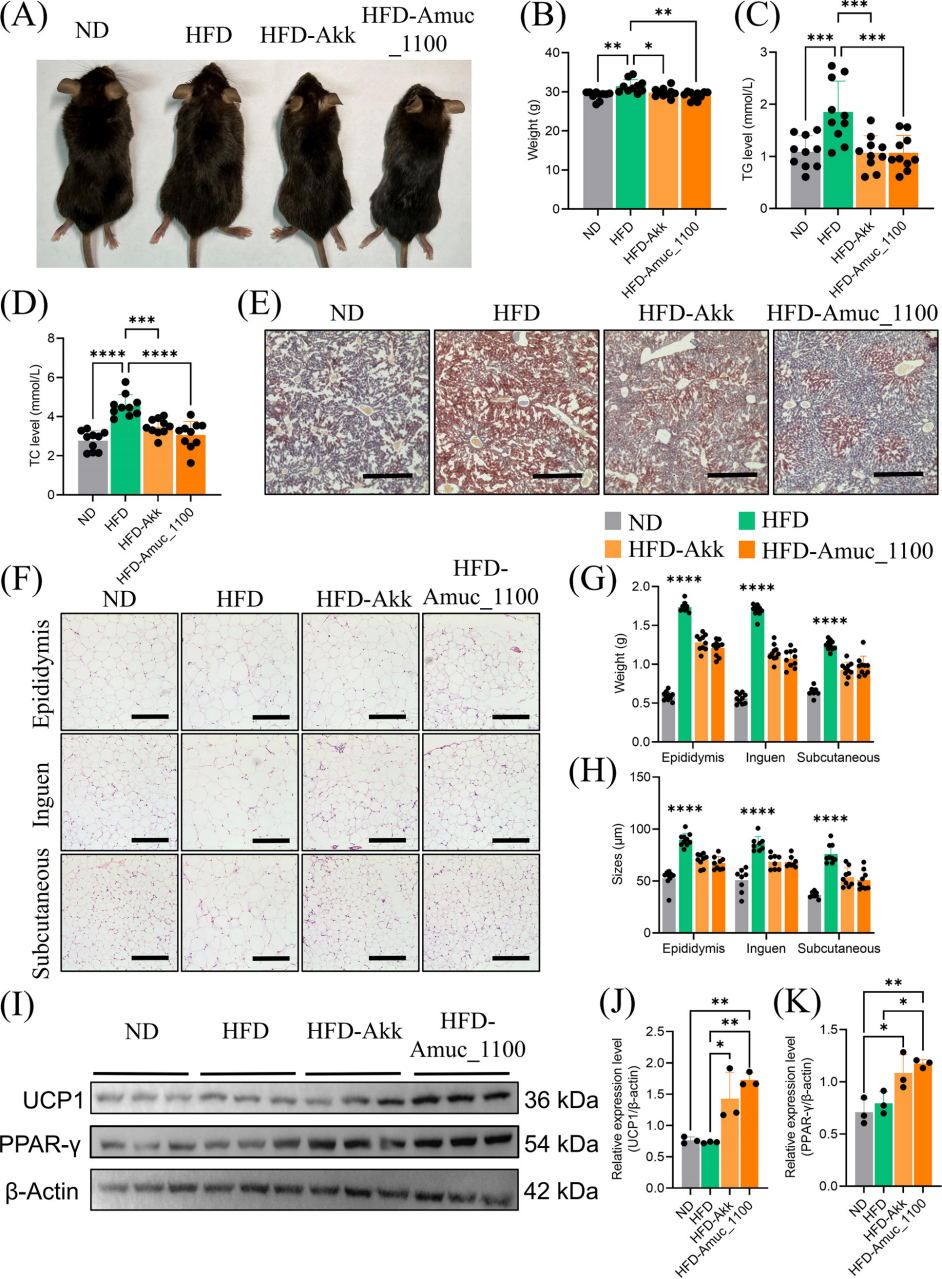
Outer membrane protein Amuc_1100 ameliorated HFD-induced obesity. (A) Representative pictures of mice. (B) Final average body weight of mice in each group. (C and D) Levels of serum triglycerides (C) and total cholesterol (D) were quantified using the cholesterol and triglyceride kits. (E) Oil Red O staining of liver sections; scale bars, 200 μm. (F) Representative images of H&E-stained sections of epididymal adipocytes, inguinal adipocytes, and subcutaneous adipocytes; scale bars, 200 μm. (G) The weights of epididymal adipocytes, inguinal adipocytes, and subcutaneous adipocytes. (H) The diameters of epididymal WAT (eWAT), inguen WAT (iWAT), and subcutaneous WAT (sWAT) were quantified by Image J software. Data are presented as mean ± SEM with *n* = 8 to 10 in each group. (I to K) Representative Western blots of WAT lysates in ND and HFD groups. Data are presented as the mean ± SEM with *n* = 3 in each group. A one-way ANOVA coupled with a Tukey multiple-comparison test was used for multiple comparisons; *, *P* < 0.05; **, *P* < 0.01; ***, *P* < 0.001.

To explore the effect of Amuc_1100 on the progression of obesity, C57BL/6 mice were randomly divided into 4 groups, including 2 control groups (normal diet [ND] or high-fat diet [HFD]) and 2 experimental groups (HFD-Akk or HFD-Amuc_1100). A HFD combined with Akk at a dose of 5 × 10^9^ CFU/200 μL of phosphate-buffered saline (PBS; HFD-Akk) or the protein Amuc_1100 (HFD-Amuc_1100) at a dose of 5 μg in 200 μL of PBS was given daily to the mice by oral administration for 12 weeks. Control groups fed with a ND or HFD alone were treated with PBS. Compared with the HFD control group, both Amuc_1100 (*P* < 0.01) and Akk (*P* < 0.05) noticeably diminished average total weight in HFD-induced mice (Fig. 1A and B). Moreover, we found that administration of Akk or Amuc_1100 protein reduced levels of serum triglycerides (TG; *P* < 0.01) and total cholesterol (TC; *P* < 0.01) in the HFD cohort (Fig. 1C and D). Oil Red O staining demonstrated that Amuc_1100 and Akk significantly decreased lipid accumulation in HFD mouse livers (Fig. 1E). Importantly, a high-fat diet increased adipose tissue weights and adipocyte sizes compared to the ND; however, these measures were both decreased after Akk and Amuc_1100 supplementation (Fig. 1F to H). Western blotting was performed, and the results revealed that HFD-Akk and HFD-Amuc_1100 mice exhibited remarkably increased protein expression levels of browning marker genes *UCP1* and *PPAR-γ* (Fig. 1I to K). The results indicate that Amuc_1100 replicates the effect of Akk on browning of white adipose tissue (WAT).

### Effects of Amuc_1100 on lipogenesis in 3T3-L1 preadipocytes.

**FIG 2:**
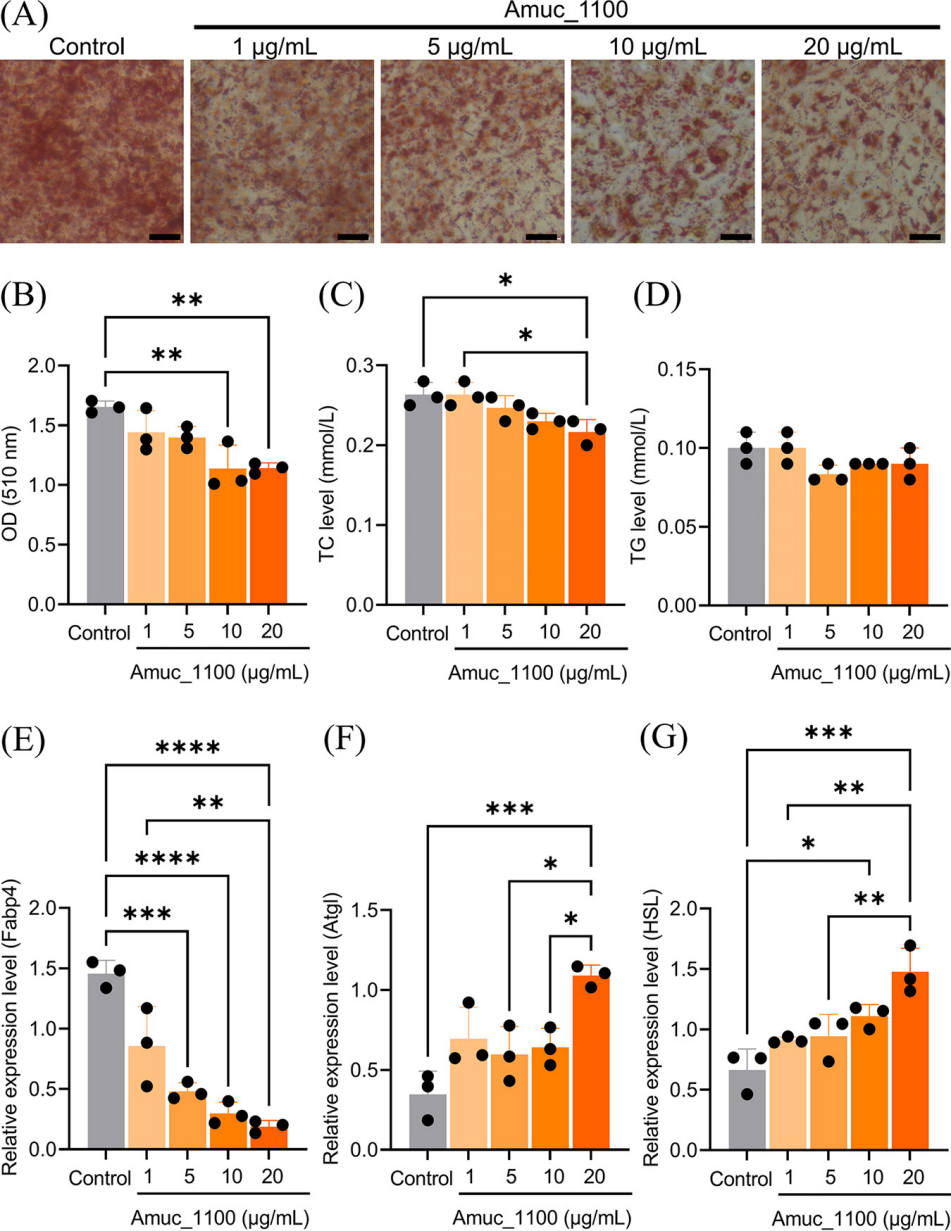
Amuc_1100 inhibited lipogenesis and lipid accumulation in 3T3-L1 induced white adipocytes. (A) Oil Red O staining of the treated cells and the controls on day 6 after induction of differentiation. Images were captured at ×40 magnification; scale bars, 100 μm. (B) Lipid content was assessed by measuring the absorbance of the Oil Red O eluted from 3T3-L1 adipocytes at a 510-nm wavelength. (C and D) Total extracellular levels of cholesterol (C) and triglycerides (D) in 3T3-L1 adipocytes were quantified using the cholesterol and triglyceride kits. (E to G) mRNA expression levels of the gene *Fabp4* (E) and genes *Atgl* (F) and *HSL* (G) normalized to 18S rRNA are shown. Data are presented as mean ± SEM (*n* = 3) and were compared to controls; *, *P* < 0.05; **, *P* < 0.01; ***, *P* < 0.001.

To investigate the effect of Amuc_1100 on lipid formation and accumulation, we induced adipocyte differentiation in 3T3-L1 cells for 6 days. The viability assay CCK8 showed that Amuc_1100 did not affect cell viability and proliferation (Fig. S1 in the supplemental material). Oil Red O staining and absorbance measurements showed that, compared with the control group, Amuc_1100 treatment reduced adipogenesis and the accumulation of intracellular lipids (Fig. 2A and B). Moreover, we found that total cholesterol (TC) levels decreased in 3T3-L1 cell medium treated with Amuc_1100 at different concentrations, and the concentration of Amuc_1100 at 20 μg/mL had the most significant effect. However, levels of another major lipid component, triglyceride (TG), in 3T3-L1 cell culture medium treated with different concentrations of Amuc_1100 had no statistical difference but showed a declining trend (Fig. 2C and D). To further evaluate the impact of Amuc_1100 on lipid metabolism in 3T3-L1 preadipocytes, the mRNA expression levels of genes involved in lipogenesis and lipolysis were measured. As a key lipase in fat mobilization, the role of Atgl in adipose tissue is inextricably linked to HSL function (21). As shown in Fig. 2E, Amuc_1100-treated cells showed a significant decrease in the mRNA expression levels of one lipogenesis gene (*Fabp4*). In contrast, the mRNA expression levels of two important lipolysis-related genes, adipose triglyceride lipase (*Atgl*) and hormone-sensitive lipase (*HSL*), were promoted in the Amuc_1100 groups (Fig. 2F and G). These data suggest that Amuc_1100 demonstrates the ability to inhibit adipogenesis and lipid accumulation in 3T3-L1 preadipocytes.

### Effects of Amuc_1100 on browning and thermogenesis of 3T3-L1 preadipocytes.

**FIG 3:**
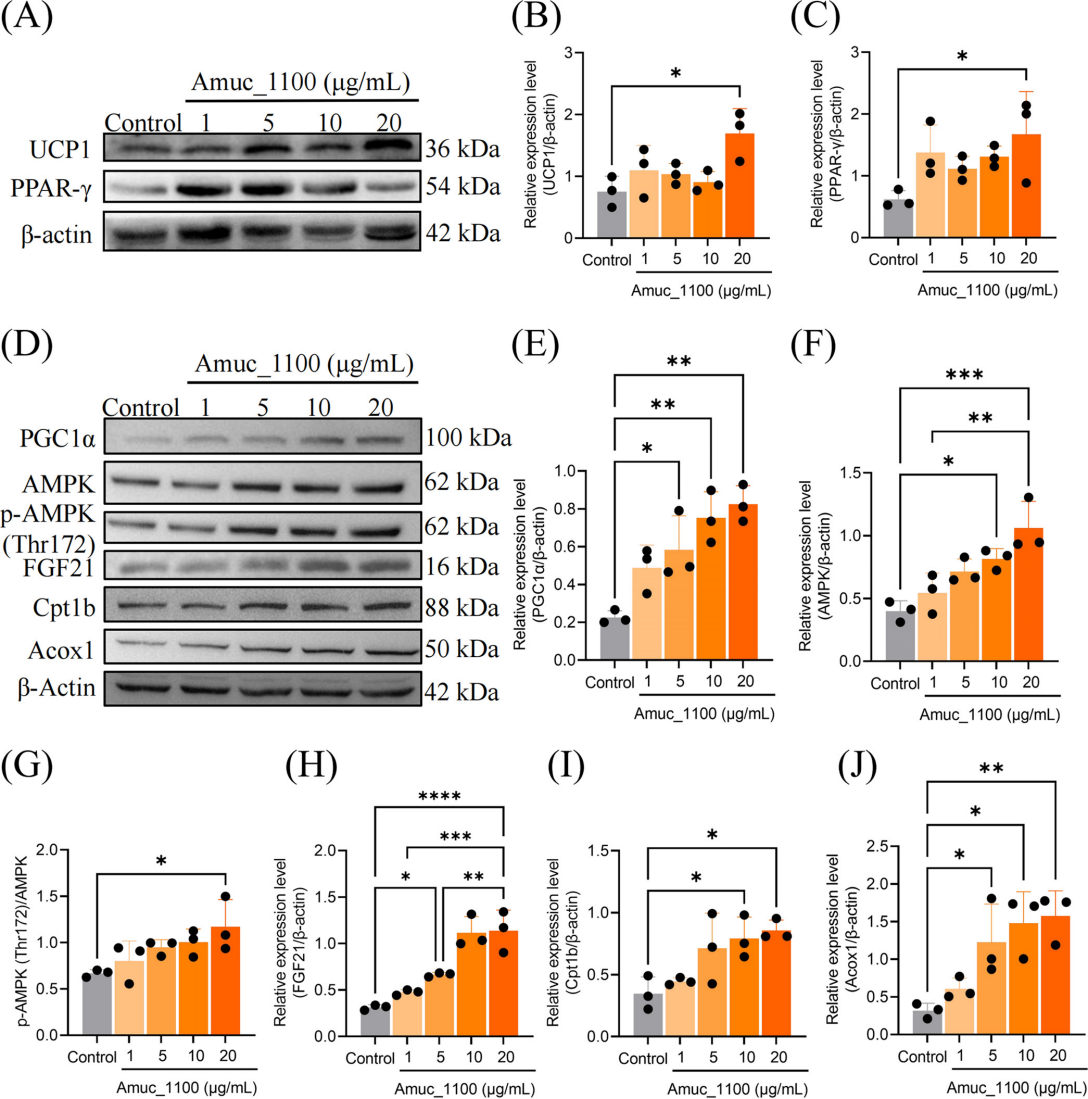
Amuc_1100 improved thermogenic and lipolysis gene expression in 3T3-L1 cells. Cells were treated with Amuc_1100 at concentrations of 1, 5, 10, and 20 μg/mL for 4 days. Protein expression levels of UCP-1 (A and B) and PPAR-γ (A and C) are shown for the treated cells and the controls. (D) Representative Western blot of cell lysate. Expression levels of PGC1α (E), AMPK (F), p-AMPK (G), FGF21 (H), Cpt1b (I), and Acox1 (J) in 3T3-L1 cells after incubation with Amuc_1100. Data are expressed as mean ± SEM (*n* = 3); *, *P* < 0.05; **, *P* < 0.01; ***, *P* < 0.001.

To determine whether Amuc_1100 modulates the browning and thermogenesis process of 3T3-L1 preadipocytes, we measured expression levels of brown adipocyte marker proteins and genes of preadipocytes at the end of adipocyte differentiation. The 3T3-L1 cells were cultured for 6 days with an inducing medium and were supplemented with Amuc_1100 at day 3. Total proteins were extracted, and the expression of browning-specific genes was evaluated by Western blotting. As shown in Fig. 3A to C, compared to the controls, Amuc_1100-treated cells exhibited increased protein expression levels of thermogenic and beige adipocyte marker genes *UCP1* and *PPAR-γ*. Accordingly, Amuc_1100 treatment significantly increased the protein expression of peroxisome proliferator-activated receptor-γ (PPAR-γ), coactivator-1α (PGC1α) (Fig. 3D and E), and its upstream regulator AMPK (Fig. 3D and F), a metabolic regulator with beneficial effects on mitochondrial biogenesis. In addition, the treatment of Amuc_1100 increased the phosphorylation of AMPK at Thr 172, which reflects AMPK activity (Fig. 3D and G). Meanwhile, cells incubated with Amuc_1100 displayed a higher protein expression level of FGF21 (Fig. 3D and H), which promotes thermogenic activity by posttranscriptionally increasing PGC1α protein expression in adipose tissue. Analyzing the effect of Amuc_1100 on lipid metabolism, important genes encoding enzymes associated with the fatty acid beta-oxidation pathway were measured. As shown in Fig. 3D, I, and J, Amuc_1100 treatment induced increased protein expression of Cpt1b and Acox1, confirming the ability of Amuc-1100 to promote thermogenesis from lipolysis. Consistent with protein expression levels, mRNA expression levels showed similar changes in *PGC1α*, *AMPK*, *FGF21*, *Cpt1b*, and *Acox1* (Fig. S2). Taken together, these data show that Amuc_1100 promotes thermogenesis and white-to-brown adipocyte conversion.

### Mechanism of Amuc_1100 to accelerate lipolysis.

**FIG 4:**
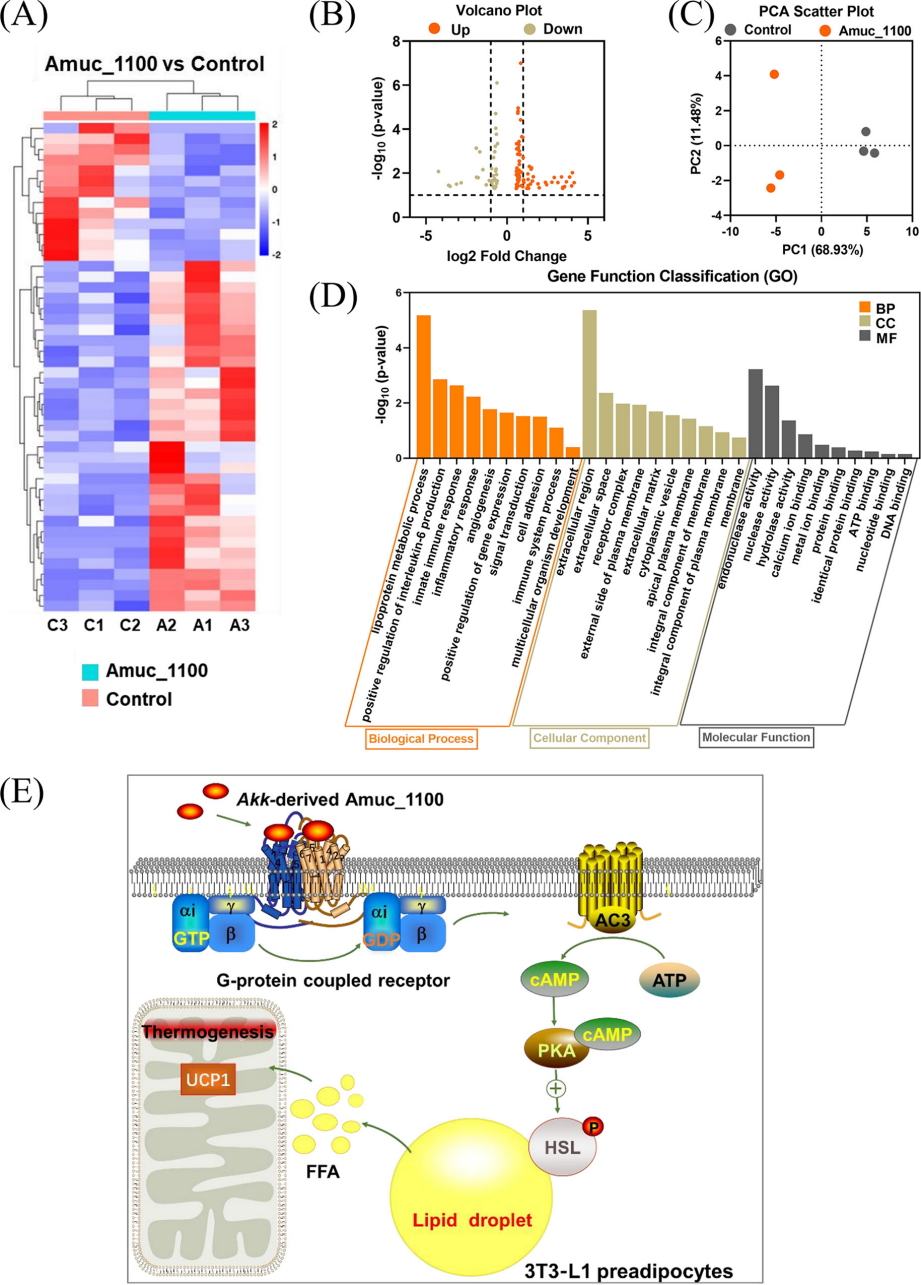
Amuc_1100 regulated lipid metabolism through the AC3/PKA/HSL pathway. (A) Heatmap of differentially expressed genes (DEGs) from Amuc_1100-treated 3T3-L1 cells versus control cells (*n* = 3 for each group, fold change > |2|, false-discovery rate [FDR] < 0.05). (B) DEGs were analyzed by volcano plot between the two groups. (C) PCA analysis. (D) GO enrichment analysis of the DEGs; BP, biological process; CC, cellular component; MF, molecular function. (E) KEGG analysis of the target pathway.

To identify how Amuc_1100 suppresses fat accumulation, transcriptomics based on an RNA-sequencing (RNA-seq) assay was used to investigate the differences in gene expression between Amuc_1100-treated samples and control cells. The main differentially expressed genes (DEGs) are presented in Fig. 4A and B. We conducted a principal-component analysis (PCA) from different dimensions to evaluate the biological variability of gene transcription between Amuc_1100-stimulated cells and the controls. The PCA plots showed that the two groups were significantly distinguished by the two principal components, accounting for 68.93% and 11.48% of the variation, respectively (Fig. 4C). To identify the biological functions and signaling pathways associated with lipid metabolism, the DEGs were used to conduct Gene Ontology biological process (GO-BP) enrichment and KEGG pathway analyses. As shown in Fig. 4D, the results showed that these DEGs were enriched in the GO biological terms performing functions in biological processes related to lipoprotein metabolic processes, regulation of interleukin-6 production, and the innate immune response, implying that Amuc_1100 may alleviate disorders of lipid metabolism and inflammatory responses associated with obesity. KEGG pathway analysis of key genes identified metabolic and thermogenesis pathways (*AC3*, *PKA*, and *HSL*), which are critical determinants of lipid decomposition, suggesting that Amuc_1100 is likely to promote lipolysis by acting on the AC3/PKA/HSL signaling pathway (Fig. 4E).

### Amuc_1100 promotes lipolysis by improving the phosphorylation of HSL.

**FIG 5:**
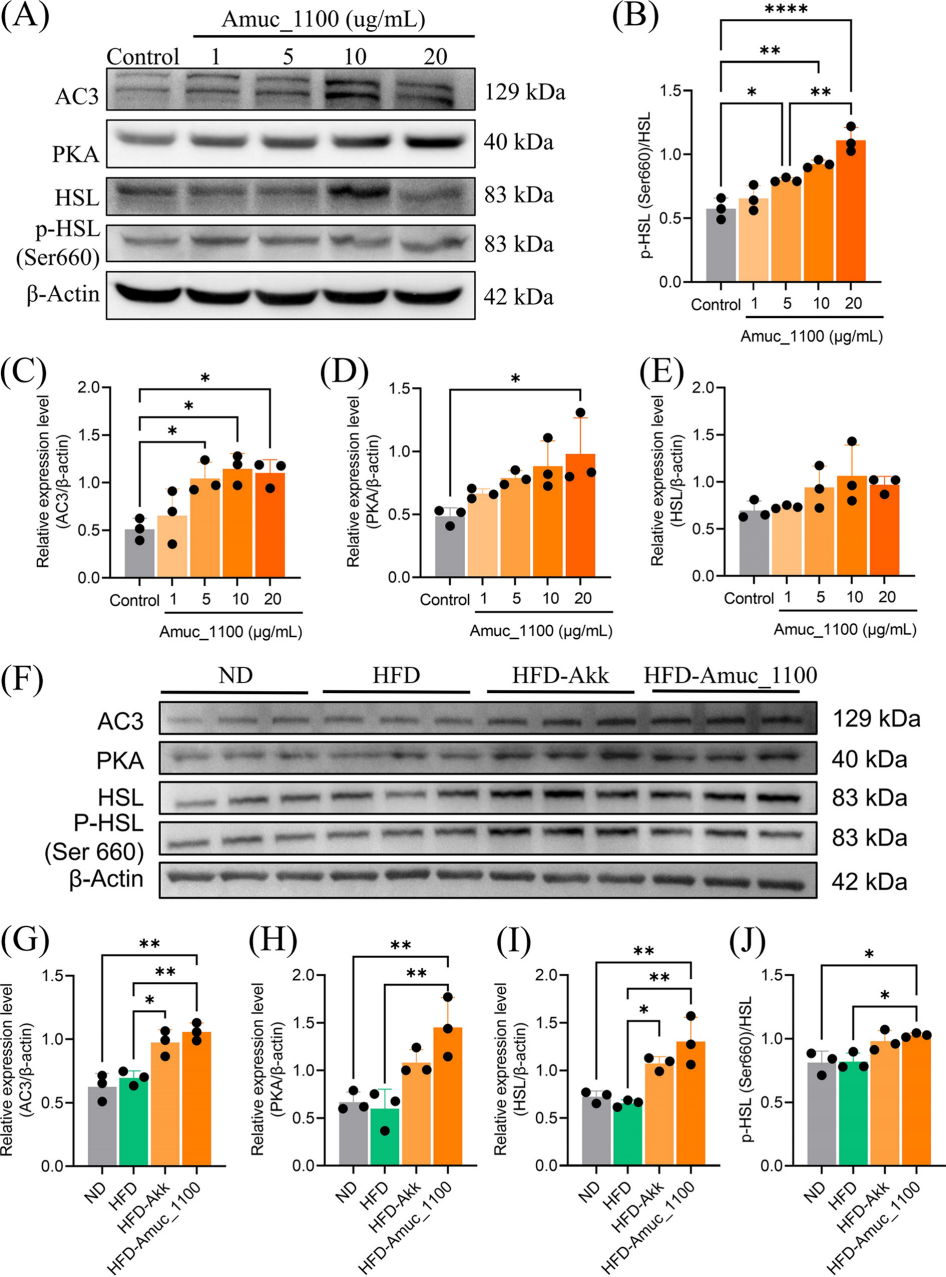
Amuc_1100 promotes Ser 660 phosphorylation of HSL. (A and F) Effects of Amuc_1100 on the protein expression of AC3, PKA, HSL, and phospho-HSL Ser 660 (p-HSL) in 3T3-L1 preadipocytes and WAT from mice were evaluated by Western blotting. β-Actin served as a loading control. (B and J) The gray value ratio of phosphorylated protein p-HSL Ser 660 to total HSL. (C to E and G to I) The gray value ratio of target proteins to β-actin (*n* = 3; *, *P* < 0.05; **, *P* < 0.01; ***, *P* < 0.001).

It is well known that β-adrenergic and growth factors stimulate the activation of adenylate cyclase (AC3), which acts on protein kinase A (PKA) and is involved in adipose tissue lipolysis. HSL, the major effector of lipolysis, is the downstream target of PKA (Fig. 4E) (22, 23). Therefore, Western blotting was used to determine the effects of Amuc_1100 on the expression levels of different proteins and genes known to be related to lipolysis *in vivo* and *in vitro* (Fig. 5A and F). Our results demonstrated that Amuc_1100 treatment significantly promotes the protein expression level of HSL (Fig. 5E and I) and its upstream signaling factors, including AC3 (Fig. 5C and G) and PKA (Fig. 5D and H), in 3T3-L1 preadipocytes and WAT of mice with Amuc_1100. Furthermore, Amuc_1100 increased the phosphorylation level of HSL to an extent in 3T3-L1 preadipocytes (Fig. 5B) and subcutaneous white adipose tissue (Fig. 5J). These data suggest that Amuc-1100 produces the lipolysis effect by regulating phosphorylation levels of HSL to promote lipolysis.

## DISCUSSION

The prevalence of obesity around the world causes a heavy health burden, and obesity is a high-risk factor for diabetes, nonalcoholic steatohepatitis, and various tumor types (1–5). The development of obesity is linked to lipid accumulation in adipocytes (6). Additionally, many studies have proposed that the progression of obesity is correlated with intestinal microflora dysbiosis in humans and mice (11–13). As a beneficial intestinal symbiont, studies have shown that Akkermansia muciniphila (Akk) contributes to improving carbohydrate and lipid metabolism, but the mechanism of its regulation in adipocytes has not yet been elucidated. We previously reported that supplemental Akk relieved hyperlipidemia and reduced liver fat accumulation in HFD-fed mice (24, 25). Moreover, to the best of our knowledge, the present study is the first to explore the specific components of Akk conveying the inhibitory effect on lipid accumulation in adipocytes. Our work found that intervention with the Akk membrane protein Amuc_1100 remarkably inhibited adipocyte differentiation of 3T3-L1 preadipocytes, significantly reduced fat synthesis and deposition, and improved lipolysis and thermogenesis.

Amuc_1100 administration reduced lipid synthesis by hindering key genes to initiate preadipocyte differentiation and adipogenesis. Our study found that in the presence of Amuc_1100, 3T3-L1 preadipocytes demonstrated markedly decreased mRNA expression levels of *Fab4*. Consequently, the inhibition of *Fab4* alleviated lipid synthesis and accumulation.

Amuc_1100 accelerated browning and thermogenesis of 3T3-L1 preadipocytes by upregulating thermogenesis-related transcription factors and their downstream target genes. Amuc_1100 administration significantly increased the relative mRNA expression of *PPAR-γ*, *PGC1α*, and the *PGC1*α posttranscriptional regulatory factor *FGF21*, thus inducing elevation of the downstream target *UCP1*. This process promoted fatty acid oxidation metabolism and browning of preadipocytes (26). These findings suggest that Amuc_1100 induces adaptive *UCP1*-mediated thermogenesis and energy expenditure by positively modulating the major regulators of *UCP1* (*PPAR*-γ and *PGC1*α) in 3T3-L1 preadipocytes.

Supplementation with Amuc_1100 might promote lipid oxidation by activating phosphorylation of HSL through the AC3/PKA/HSL pathway, greatly reducing lipid accumulation in adipocytes. Together, these experiments provide novel insight into the role of Amuc_1100 in lipid expenditure. Our study showed that Amuc_1100 activates adenylyl cyclase (AC3) in a dose-dependent manner in 3T3-L1 preadipocytes. Moreover, Amuc_1100 causes an increase in the expression of PKA and enhanced the phosphorylation of HSL at Ser 660. Phosphorylation of HSL efficiently catalyzed the hydrolysis of triacylglycerol into glycerol and free fatty acids. These data suggest that Amuc_1100-induced upregulation of phospho-HSL (p-HSL) was mediated by activation of AC3/AMPK/PKA signaling, thus enhancing lipid decomposition and thermogenesis.

However, the mechanism as to how Amuc_1100 increases adipose triglyceride lipase (Atgl) has remained unknown. As a key lipase in fat mobilization, the role of Atgl in adipose tissue is inextricably linked to HSL function (27). Thus, further studies are necessary to fully understand the effect of Amuc_1100 on lipolysis activated by Atgl. In addition, inflammation is tightly associated with lipolysis, and interleukin-6 (IL-6) was proven to induce lipolysis and attenuate the insulin response. We found that Amuc_1100 intervention significantly reduced the mRNA levels of *Il6* in 3T3-L1 cells (Fig. S1 in the supplemental material), demonstrating that Amuc_1100 inhibits inflammation-associated lipolysis and indicating that Amuc_1100 protects the body from metabolic syndromes and has less adverse effects.

Amuc_1100, an important functional protein of Akk, is located on the outer membrane and recapitulates the effects of the bacterium on adipocytic differentiation and lipid metabolism (20). However, it remains unknown how Akk delivers its outer membrane protein to adipose tissues and exerts the above functions. Recent studies found that extracellular vesicles (EVs) are spherical nanostructures derived from the lipid membranes of the cell surface, which are composed of a cavity sphere with lipid bilayers ranging from 20 nm to 500 nm in diameter (28, 29). EVs originating from the bacterial outer membrane are therefore often referred to as bacteria extracellular vesicles (BEVs). BEVs can carry different cargo, including nucleic acids, adhesins, virulence factors, immune regulatory factors, and so on (30, 31). Previous studies have revealed that BEVs can interact with the host and other microbiota and play different roles (32, 33). We were, therefore, curious to determine in the future whether Akk EVs could carry the membrane protein Amuc_1100 to adipose tissues and exert a lipolysis effect, which has important implications for the development of new tools for the treatment of obesity. In addition, further studies are needed to confirm the therapeutic effects of Amuc_1100 in humans.

### Conclusions.

In conclusion, our work demonstrated that Amuc_1100 is the bioactive fraction responsible for the antiobesity activity of Akk. Amuc_1100 appears to stimulate the AC3/PKA/HSL pathway, thus enhancing lipolysis. Our study provides a foundation for elucidating the mechanism of Akk in alleviating obesity and related metabolic disorders and offers a new idea for obesity therapy.

## MATERIALS AND METHODS

### Production of Amuc_1100 protein.

Amuc_1100 protein was purchased from Nanjing Kingsray Biotechnology Co., Ltd., and was produced according to a previous study (34). The PCR product of a His-tagged Amuc_1100 was cloned into the pET-22b (+) vector (GenScript, Nanjing, China) to construct the expression plasmid, which was validated by visualizing NdeI- and XhoI-digested fragments on an agarose gel. The following primer sequences were used for the construct: **5′-****GGGTACCATATGATCGTCAATTCCAAACGC****-3′** (forward) and **5′-****CCTTGGCTCGAGATCTTCAGACGGTTCCTG****-3′** (reverse). Bolded sequences are restriction sites for NdeI and XhoI enzymes, respectively (Thermo Scientific, MA, USA). The plasmid pET-22b-Amuc_1100 was transformed into Escherichia coli BL21(DE3), which was cultured in LB broth with kanamycin (50 μg/mL) with shaking at 220 rpm at 37°C. Isopropyl-β-d-1-thiogalactopyranoside (IPTG; 1 mM) was added to the LB broth to induce Amuc_1100 expression during the midexponential phase. The bacteria were harvested by centrifuging for 10 min at 5,000 × *g* when the optical density at 600 nm (OD_600_) reached 1.0, and the cell pellets were then resuspended and lysed using lysozyme and sonification (Sonifier 450, Branson Ultrasonics Corporation, Danbury, CT, USA); the centrifugal supernatant was used to purify the Amuc_1100 protein by metal affinity purification under native conditions using Ni-16NTA His•Bind resin (Novagen, Merck Millipore, MA, USA). The concentration of Amuc_1100 was determined by a bicinchoninic acid (BCA) protein assay kit (Beyotime Biotechnology, China). Amuc_1100 protein was stored at −80°C until use.

### Culture and administration of Akkermansia muciniphila.

Akkermansia muciniphila (ATCC, BAA-835) was cultured anaerobically in brain heart infusion (BHI) broth (Oxoid, England) supplemented with 0.5% porcine mucin (Sigma, Germany). After 48 h of culture, the medium was centrifuged for 10 min at 3,000 rpm. The remaining supernatant was removed, and the precipitate was resuspended with sterile PBS and centrifuged again at 3,000 rpm for 10 min. The supernatant was again removed, and the pellet was resuspended in sterile PBS.

### Animal studies.

Forty male C57BL/6 mice were purchased from the Medical Experimental Animal Center of Guangdong Province (Guangdong, China; Guangdong Medical Laboratory Animal Center, Guangzhou, China) at 7 weeks of age and were randomly and blindly divided into 4 groups, with an average of 10 animals in each group. The animal study was approved by the Institutional Animal Care and Use Committee (IACUC) of Southern Medical University (approval number SMUL2022002). After a 1-week acclimation period (mice were fed a normal chow diet after arrival), mice were fed a chow diet (Guangdong Medical Laboratory Animal Center, Guangzhou, China), a 60 kcal% fat HFD (Guangdong Medical Laboratory Animal Center, Guangzhou, China), or a HFD supplemented with Akk or Amuc_1100 for 12 weeks. After this period, the mice were starved overnight and euthanized, and blood and tissue samples were collected. The livers and adipose tissues were removed, rinsed with physiological saline, weighed, immediately frozen in liquid nitrogen, and stored at −70°C until analysis. Serum levels of TG and total cholesterol (TC) were measured using kits supplied by Abbott on an automatic biochemistry analyzer C16000 (Abbott C16000, Abbott Park, IL, USA) using the manufacturer’s protocols.

### Morphology of mouse liver and adipose tissues.

The liver, inguen WAT (iWAT), subcutaneous WAT (sWAT), and epididymis WAT (eWAT) were removed from each mouse and prepared and stained with hematoxylin and eosin (H&E) and Masson’s trichrome (MT). Mouse liver and adipose tissues were fixed with 4% paraformaldehyde for 24 h at room temperature and processed for paraffin embedding. All slices (5 μm) were dewaxed with xylene for 10 min and sequentially immersed with anhydrous ethanol for 10 min, 90% ethanol for 5 min, 85% ethanol for 5 min, and 75% ethanol for 5 min. Paraffin sections were cut and evaluated using H&E staining. Image acquisition was performed using a microscope (Olympus America, Inc., Melville, NY, USA). Adipocyte cross-sectional area and distribution were determined using ImageJ software (NIH image software). For lipid staining, Oil Red O staining was performed in liver tissues. Samples were fixed at room temperature with 4% paraformaldehyde for 24 h, dehydrated, embedded with optimal cutting temperature (OCT) compound, and sliced using the freeze slicer. The slices were stained at 37°C with Oil Red O (Servicebio, China) solution for 15 min using the Lipid (Oil Red O) staining kit from Biovision according to the manufacturer’s instructions. Nuclei were counterstained with hematoxylin for 10 min, mounted with glycerol, and examined under a light microscope. The diameters of inguinal adipocytes and epididymal adipocytes were quantified by Image J software. ImagePro Plus 6.0 software was used to analyze the number of colonic mucus cells and the degree of liver fat accumulation.

### Cell culture.

The 3T3-L1 preadipocyte cells were purchased from ATCC (Manassas, VA, USA) and cultured in Dulbecco’s modified Eagle’s medium (DMEM; Sigma-Aldrich, St. Louis, MO, USA) supplemented with 10% fetal bovine serum (FBS; Invitrogen, Carlsbad, CA, USA) and 1% penicillin-streptomycin (Invitrogen). Cells were maintained in a humidified incubator at 37°C with 5% CO_2_.

### Cell differentiation.

When cultured 3T3-L1 preadipocytes reached 70 to 80% confluence, differentiation was induced using 500 μmol/L 3-isobutyl-1-methylxanthine (Sigma-Aldrich), 1 μmol/L dexamethasone (Sigma-Aldrich), and 10 μg/mL insulin differentiation medium. Cells were induced to differentiate by transferring to differentiation medium for 48 h. After 2 days, the differentiation medium was replaced with a routine culture medium consisting of 10% FBS, 10 μg/mL insulin, and DMEM (maturation medium). The medium was changed every 48 h for 4 days. Cells were grouped into the control and model groups. For Amuc_1100 treatment, cells were maintained in maturation medium with various concentrations of Amuc_1100 protein (1, 5, 10, and 20 μg/mL), followed by differentiation for 96 h until mature adipocyte formation and analysis.

### Oil Red O staining.

The Oil Red O staining assay was used to detect lipid droplets. Completely differentiated 3T3-L1 preadipocyte cells were washed with phosphate-buffered saline (PBS), fixed with 4% paraformaldehyde for 1 h at room temperature, and washed 3 times with deionized water. Cells were stained with 100 μL of fresh Oil Red O working solution, incubated at room temperature for 20 min, and rinsed 3 times with deionized water. An inverted light microscope was used to observe the cell staining. Intracellular lipid content was quantified by eluting Oil Red O bound to cells with 100% isopropanol and measuring its absorbance at 510 nm on a microplate reader (BioTek, USA).

### Biochemical analysis.

The levels of TG and TC in sera and cell media were measured using an Abbott Automatic Biochemistry Analyzer C16000 and its accompanying reagents (Abbott C16000, Abbott Park, IL, USA).

### RNA isolation and real-time qPCR analysis.

The primer sequences of lipid metabolism, brown adipose tissue (BAT) markers, and inflammation genes are shown in Table S1. Total RNA was extracted from the 3T3-L1 induced adipocytes using TRIzol reagent (Beyotime, China). The concentration and purity (OD_260_/OD_280_) of RNA were determined on a NanoDrop 2000c spectrophotometer (Thermo Fisher Scientific, Waltham, MA, USA). cDNA was prepared using a TaKaRa reverse transcription reagent kit (TaKaRa, Japan). Relative expression of the target mRNA was detected by PCR using qPCR SYBR green master mix (Applied Biosystems, Waltham, MA, USA) and performed on a 7300 Fast real-time PCR system (Applied Biosystems). Data were analyzed using the cycling threshold (2^−ΔΔ^*^CT^*) method.

### Western blotting.

Protein samples from 3T3-L1 adipocytes were obtained using RIPA lysis buffer containing 1 mM phenylmethanesulfonyl fluoride (PMSF; FDbio, China). The total protein concentration was determined using the BCA method (FDbio, China). Equal amounts of proteins (40 μg per group) were loaded for SDS-PAGE electrophoresis. After transferring protein to polyvinylidene difluoride (PVDF) membranes, the blots were blocked with 5% skim milk for 1 h at room temperature. The membranes were incubated with primary antibodies overnight at 4°C and corresponding secondary antibodies conjugated with anti-rabbit IgG conjugated to horseradish peroxidase for 2 h at room temperature. All antibodies were obtained from Abcam (Waltham, MA, USA). β-Actin was used for normalization. Bands were visualized using a chemiluminescence kit (Bio-Rad, Hercules, CA, USA) and quantified using ImageJ software (National Institutes of Health, Bethesda, MD, USA).

### RNA sequencing and data analysis.

The 3T3-L1 adipocytes were induced using 500 μmol/L 3-isobutyl-1-methylxanthine (Sigma-Aldrich), 1 μmol/L dexamethasone (Sigma-Aldrich), and 10 μg/mL insulin differentiation medium for 48 h and replaced with a routine culture medium consisting of 10% FBS, 10 μg/mL insulin, and DMEM (maturation medium). After the medium was changed every 48 h for 4 days, cells were maintained in maturation medium with various concentrations of Amuc_1100 protein (20 μg/mL), differentiated for 96 h until mature adipocyte formation, and used for transcriptome analysis. Total RNA was extracted using the mirVana miRNA isolation kit (Ambion, Thermo Fisher Scientific) following the manufacturer’s protocol. RNA integrity was evaluated using the Agilent 2100 Bioanalyzer (Agilent Technologies, Santa Clara, CA, USA). Samples with RNA integrity numbers (RINs) of ≥7 were subjected to subsequent analysis. The libraries were constructed using a TruSeq stranded mRNA LTSample prep kit (Illumina, San Diego, CA, USA) according to the manufacturer’s instructions. These libraries were sequenced on an Illumina sequencing platform (HiSeq 2500 or Illumina HiSeq X 10), and 125-bp/150-bp paired-end reads were generated. The clean reads were mapped to the mouse genome (GRCm39) using HISAT2. Fragments per kilobase per million (FPKM) values of each gene were calculated using Cufflinks, and the read counts of each gene were obtained by HTSeq-count. The heatmap and volcano plot were generated using R 4.1.1 software to identify differentially expressed genes. *P* values of <0.05 and fold change values of >2 were set as the threshold for significant differential expression. A principal-component analysis (PCA) was conducted using the R “prcomp” function. GO enrichment and KEGG pathway enrichment analyses of DEGs were performed using R based on the hypergeometric distribution.

### Statistical analysis.

All analyses were performed using GraphPad Prism 9 software (GraphPad Software, Inc., San Diego, CA, USA). Data were expressed as the mean ± standard error of the mean (SEM) from no less than 3 separate repeated experiments. One- or two-way analysis of variance (ANOVA) with Tukey’s multiple-comparison test or a paired Student’s *t* test was performed for data analysis. All tests were two-tailed, and *P* values of <0.05 were considered to be significant.
