# Supplementary material for: Membrane Protein Amuc_1100 Derived from Akkermansia muciniphila Facilitates Lipolysis and Browning via Activating the AC3/PKA/HSL Pathway
Source: Microbiol Spectr. 2023 Feb 27;11(2):e04323-22. doi: 10.1128/spectrum.04323-22 (PMC10100790; doi:10.1128/spectrum.04323-22)
Supplement: Supplemental file 1 — Supplemental material. Download spectrum.04323-22-s0001.pdf, PDF file, 0.3 MB [file spectrum.04323-22-s0001.pdf]

Membrane Protein Amuc_1100 Derived from *Akkermansia Muciniphila* Facilitates Lipolysis and Browning *via* Activating *AC3/PKA/HSL* Pathway

Xifen Zheng[1,#], Wenting Huang[1,#], Qianbei Li[3,#], Yun Chen[4], Linyan Wu[1], Yifan Dong[1], Xinyue Huang[3], Xiaojing He[3,*], Zihao Ou[3,*] and Yongzheng Peng[1, 2,*]

1 *Department of Laboratory Medicine, Zhujiang Hospital, Southern Medical University, Guangzhou, Guangdong, 510282, China*

2 *Department of Transfusion Medicine, Zhujiang Hospital, Southern Medical University, Guangzhou, Guangdong, 510282, China*

3 *Department of Laboratory Medicine, Nanfang Hospital, Southern Medical University, Guangzhou, Guangdong, 510515, China*

4 *Department of Gynaecology and Obstetrics, Nanfang Hospital, Southern Medical University, Guangzhou, Guangdong, 510515, China*

[#]These authors contributed equally to this work.

*Correspondence:

Xiaojing He, Department of Laboratory Medicine,

Nanfang Hospital, Southern Medical University, Guangzhou, Guangdong, 510515, China

Email: 649838226@qq.com

Zihao Ou, Department of Laboratory Medicine,

Nanfang Hospital, Southern Medical University, Guangzhou, Guangdong, 510515, China

Email: ozh_xnp@smu.edu.cn

Yongzheng Peng, Department of Transfusion Medicine,

Zhujiang Hospital, Southern Medical University, Guangzhou, Guangdong, 510282, China.

Email: yzpeng1981@126.com

Authors' contributions:

Xifen Zheng and Wenting Huang designed and carried out the experiments, and wrote the paper. Xiaojing He funded this project. Qianbei Li, Yun Chen, Linyan Wu, Yifan Dong and Xinyue Huang helped analyzed data. Zihao Ou and Yongzheng Peng designed the experiments, revised the paper, and approved the final version.

Gene	Forward(5'-3')	Reverse(5'-3')
PGC-1α	GCATGGTGCCTTCGCTGA	TGGCATCTCTGTGTCAACCATG
FGF21	CGTCTGCCTCAGAAGGACTC	TCTACCATGCTCAGGGGGTC
CytC	AAATCTCCACGGTCTGTTCGG	GGGTATCCTCTCCCCAGGTG
IL-6	CTGCAAGAGACTTCCATCCAG	AGTGGTATAGACAGGTCTGTTGG
β-klotho	CACTGTGGGACACAACCTGA	CCAAGCACAGAGGACATGGA
AMPK	AACTCGGCCTCACCCTGAAA	TTTGCCTTCCGTACACCTTGG
FABP4	CGACAGGAAGGTGAAGAGCATCATA	CATAAACTCTTGTGGAAGTCACGCCT
Acox1	CGATCCAGACTTCCAACATGAG	TGCCAAATTCCCTCATCTTC
CPT1b	CCAAACGTCACTGCCTAAGCT	CCAATGTCTCCATGCGGTAA
ATGL	TGACCATCTGCCTTCCAGA	GAGAGGTTGTTTCGTACCCA
HSL	GCACTGTGACCTGCTTGGT	CTGGCACCCTCACTCCATA

Table S1. The primer sequences of lipid metabolism, BAT markers and inflammation genes.

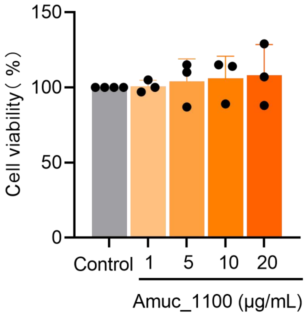

Fig. S1 Toxicity of Amuc_1100 protein on preadipocytes measured by CCK-8 method.

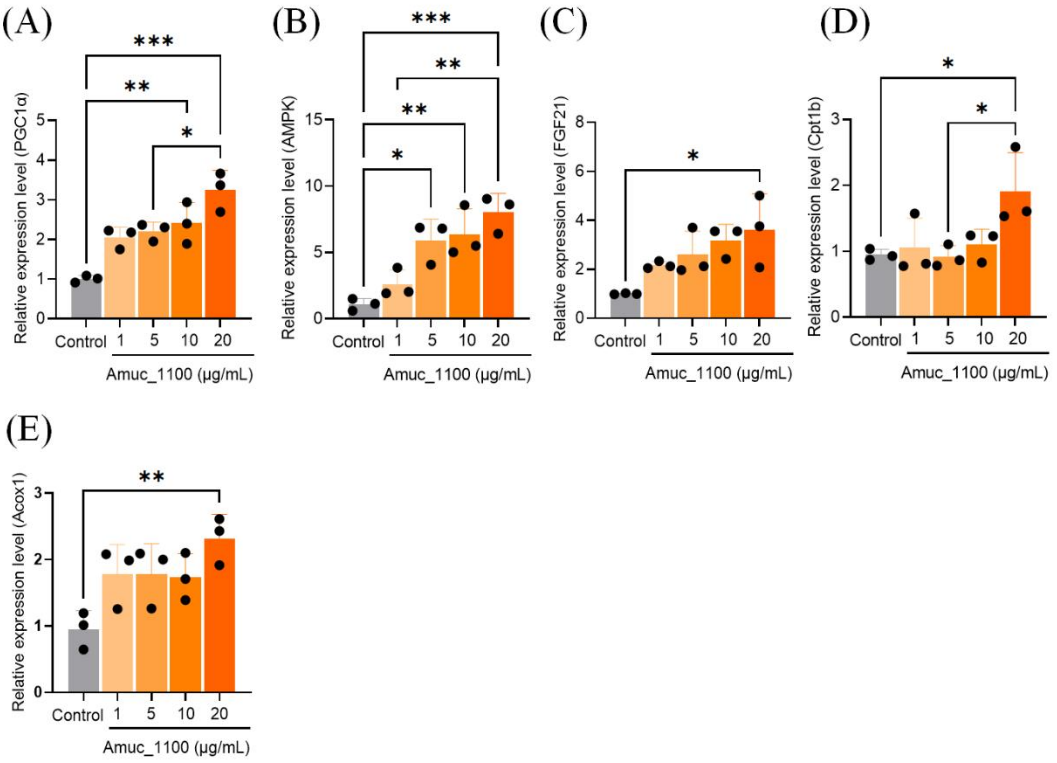

Fig. S2 Detection of mRNA levels of PGC1α (A), AMPK (B), FGF21 (C), Cpt1b (D) and Acox1 (E) in 3T3-L1 cells after incubation with Amuc_1100. Data are expressed as mean ± SEM (n=3), **P*<0.05, ***P*<0.01, ****P*<0.001; n=3.
